# Unexpected Airway Obstruction in a Patient With Undiagnosed Granulomatosis With Polyangiitis

**DOI:** 10.7759/cureus.75307

**Published:** 2024-12-08

**Authors:** Mona-Lisa Coutinho, Ricardo Portela e Silva, André Postiga, Valentina Almeida

**Affiliations:** 1 Anesthesiology, Unidade Local de Saúde de Coimbra, Coimbra, PRT

**Keywords:** airway obstruction, difficult airway, granulomatosis with polyangiitis, subglottic stenosis, vasculitis

## Abstract

Granulomatosis with polyangiitis (GPA) is a rare systemic vasculitis that can involve the respiratory tract and lead to severe airway complications. We present a 61-year-old female with an undiagnosed GPA who experienced severe airway obstruction and rapid deterioration during a flexible bronchoscopy. Severe glottic edema and reduced vocal cord mobility resulted in a challenging airway and rapid desaturation, and ultimately led to cardiac arrest. This case highlights the importance of considering GPA in the differential diagnosis of patients with unexplained respiratory symptoms, even in the absence of classic clinical features. Early diagnosis, careful pre-procedural airway assessment, and experienced airway management are essential to minimize the risk of airway compromise in patients with suspected or confirmed GPA.

## Introduction

Granulomatosis with polyangiitis (GPA), formerly known as Wegener's granulomatosis, is a rare systemic necrotizing vasculitis that predominantly affects small and medium-sized blood vessels [1]. It has an estimated incidence of 0.4 to 11.9 cases per million person-years and typically affects individuals in their fourth to sixth decades of life [2]. It is considered an autoimmune disease, strongly associated with antineutrophil cytoplasmic autoantibody (ΑNCΑ) directed against leukocyte proteinase 3 (PR3) or myeloperoxidase (MPO) [2].

While GPA can affect any organ system, the upper and lower respiratory tracts and kidneys are the most commonly and severely involved [3]. The clinical presentation is variable in terms of organ manifestations and severity, and patients often present with nonspecific prodromal symptoms, leading to initial misdiagnosis. Organ-specific manifestations of GPA can be diverse, including upper respiratory tract disease (rhinosinusitis, purulent/bloody nasal discharge, and oral and/or nasal ulcers), lower respiratory tract disease (tracheobronchial disease, lung parenchymal nodules, interstitial lung disease, and alveolar hemorrhage), renal disease (glomerulonephritis), and neurological dysfunction [4-6]. Laryngeal involvement occurs in a significant proportion of patients, with subglottic stenosis being reported in 10-20% of cases. Other forms of tracheobronchial involvement, such as bronchial ulceration and vocal cord edema, are uncommon [7].

We aim to report a case of a patient with undiagnosed GPA who presented with severe glottic edema and reduced abduction of the vocal cords during a flexible bronchoscopy. This article was previously presented as a meeting abstract at Euroanaesthesia 2024 on May 26, 2024 [8].

## Case presentation

A 61-year-old female patient was hospitalized due to suspected community-acquired pneumonia. She presented with general malaise, productive cough, pleuritic chest pain, and fever. Her medical history included hypertension, with no known allergies or relevant family history. After 5 days of hospitalization, due to persistent respiratory symptoms and inadequate response to standard antibiotic therapy, a flexible bronchoscopy under anesthesia was scheduled as part of the etiological workup.

On the day of the procedure, the patient was alert and oriented, with no signs of acute respiratory distress, including dyspnea, stridor, dysphonia, or hoarseness. Cardiopulmonary auscultation was normal. She was afebrile and hemodynamically stable (temperature: 36.4 °C, heart rate: 78 beats per minute, respiration rate: 15, blood pressure: 134/74 mmHg, and SpO_2_: 96% on 2L/min oxygen via nasal cannula). Her body mass index (BMI) was normal (weight: 66 kg, height: 164 cm). Airway assessment revealed a Mallampati class II, with no oropharyngeal abnormalities, normal mouth opening, and normal neck mobility.

**Figure 1:**
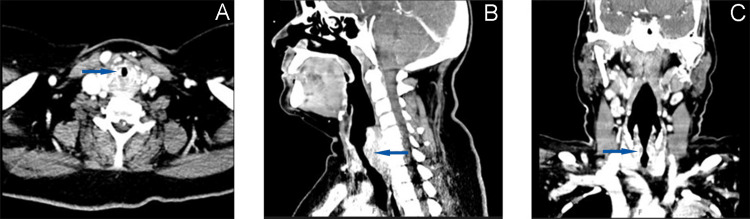
Computerized tomography images Computerized tomography images: axial (A), sagittal (B) and coronal (C) planes; subglottic and upper tracheal stenosis (blue arrows).

Laboratory tests from the same day showed mild leukocytosis, anemia, thrombocytosis, and elevated C-reactive protein (Table 1). Arterial blood gas analysis on 2L/min oxygen showed no significant abnormalities (Table 2). Blood cultures collected four days prior were negative. Computerized tomography (CT) performed two days prior revealed numerous irregular nodules and cavitations, wall thickening of the right main bronchus, and some degree of subglottic and upper tracheal stenosis (Figure 1).

**Table 1:** Blood count, metabolic panel and inflammatory markers results. BUN=blood urea nitrogen, MCV=Mean corpuscular volume, WBC=White blood cell, MCH=Mean corpuscular hemoglobin, MCHC=Mean corpuscular hemoglobin concentration, ALT=alanine transaminase, AST=aspartate aminotransferase

Parameter	Obtained Value	Reference Range
WBC count	11.8 x 10^9/L	3.9–10.2 x 10^9/L
Hemoglobin	9.5 g/dL	12.0–15.5 g/dL
Hematocrit	30.3%	35.5–45.5%
MCV	82.6 fL	80–99 fL
MCH	25.9 pg	27–33.5 pg
MCHC	31.4 g/dL	31–37 g/dL
Platelets count	444 x 10^9/L	150–400 x 10^9/L
Sodium	140 mmol/L	136–146 mmol/L
Potassium	4.4 mmol/L	3.5–5.1 mmol/L
Chloride	101 mmol/L	98–107 mmol/L
BUN	9.8 mg/dL	7.9–20.9 mg/dL
Serum creatinine	0.61 mg/dL	0.55–1.02 mg/dL
Bilirubin total	0.4 mg/dL	0.2–1.2 mg/dL
Bilirubin direct	0.1 mg/dL	0.0–0.3 mg/dL
ALT	25 U/L	< 34 U/L
AST	28 U/L	< 31 U/L
C-reactive protein	14.82 mg/dL	< 0.5 mg/dL
Procalcitonin	0.05 ng/mL	< 0.5 ng/mL

**Table 2:** Arterial blood gas analysis.

Parameter	Obtained Value	Reference Range
pH	7.43	7.35–7.45
pCO₂	39.4 mmHg	35–45 mmHg
pO₂	95.6 mmHg	83–108 mmHg
SatO₂	97.4%	94–100%
HCO₃₋	25.7 mmol/L	21–29 mmol/L
Lactate	0.4 mmol/L	0.5–2 mmol/L

The primary diagnostic hypotheses, considering both the patient's clinical presentation and the results of the complementary studies, were pneumonia, lung cancer, or a granulomatous disease.

**Figure 2:**
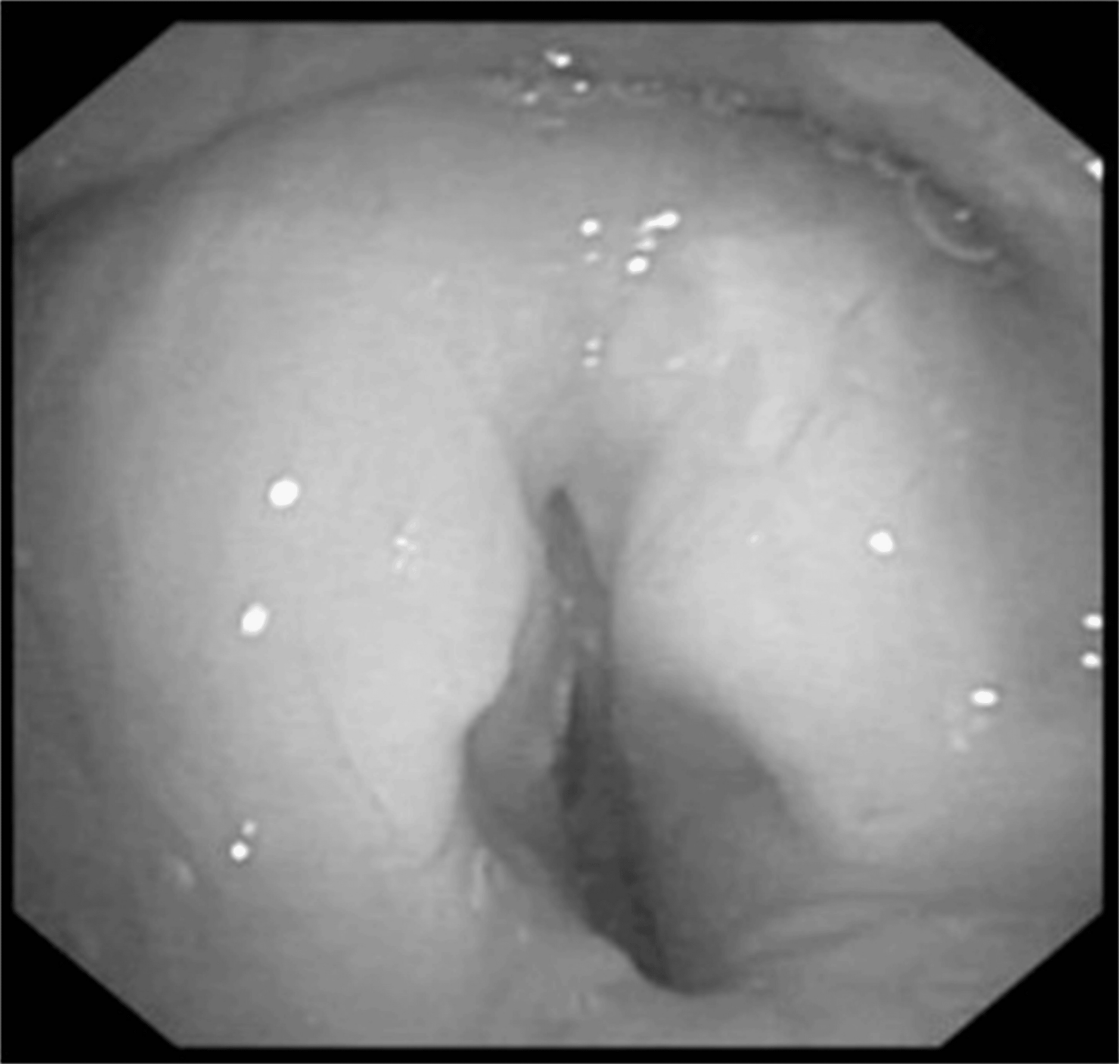
Flexible bronchoscope image Endoscopic image showing severe glottic edema and reduced vocal cord abduction.

The anesthetic plan was to induce general anesthesia and manage the airway using a supraglottic airway device, allowing for the bronchoscopy to be performed. After routine non-invasive monitoring, general anesthesia was induced with fentanyl and propofol. Difficult mask ventilation (grade 3) prompted the introduction of a second-generation supraglottic airway device (Ambu® AuraGain). Ventilation remained challenging with high peak inspiratory pressures (40-50 cmH_2_O) and irregular end-tidal carbon dioxide (EtCO_2_). To confirm the correct placement of the airway device, the flexible bronchoscope (FB) was advanced through the rubber valve-fitted opening of the angle piece. Glottis visualization revealed severe edema and reduced vocal cords abduction (Figure 2).

As the pulmonologist attempted to advance the FB through the narrowed glottis, the patient experienced rapid desaturation and a slowing heart rate. Immediate efforts were made to perform endotracheal intubation, and during this attempt, the patient progressed to asystole, leading to immediate initiation of cardiopulmonary resuscitation (CPR). The airway was secured through orotracheal intubation under direct laryngoscopy using a 6.5 mm cuffed endotracheal tube. After successful intubation, one cycle of CPR, and 1mg of adrenaline, the patient returned to spontaneous circulation.

Subsequently, the patient was admitted to the intensive care unit (ICU). One day later, the diagnosis of GPA was established with the results of the autoimmune study, which revealed a positive C-ANCA and a positive anti-PR3 titer. She was immediately started on intravenous methylprednisolone 1 g daily. After 6 days of treatment and support in the ICU, her respiratory status improved, allowing for successful extubation. Prior to extubation, a thorough airway and respiratory assessment was performed, including a cuff leak test and spontaneous breathing trial. The extubation was performed with the patient awake and spontaneously breathing. Immediately after extubation, she received supplemental oxygen 40% via Venturi mask, which was progressively reduced. The patient was discharged from the ICU one day after extubation, on 3L/min oxygen via nasal cannula without signs of acute respiratory distress or airway obstruction. She had no neurological or cardiovascular sequelae.

## Discussion

GPA is a multisystemic vasculitis with a predilection for the upper and lower respiratory tract and kidneys [1]. While the precise etiology of GPA is unknown, it is widely accepted as an autoimmune disease with approximately 92% of patients being ANCA positive [9]. Clinical presentation often involves nonspecific constitutional symptoms that may last for weeks or months without any evidence of specific organ involvement [10]. As a result, GPΑ can be frequently misdiagnosed initially as an infectious or malignant pathology. Specific organ manifestations may include rhinosinusitis, hoarseness, cough, dyspnea, stridor, hemoptysis, glomerulonephritis, and neurologic dysfunction.

The diagnosis of GPA is often challenging and requires a high index of suspicion. Clinical features, laboratory tests, and imaging studies are essential for diagnosis. Pulmonary function tests may reveal an obstructive pattern due to diffuse airway involvement, a restrictive pattern due to pulmonary fibrosis, or a mixed pattern [6]. Chest imaging, particularly high-resolution CT, plays a crucial role in the diagnostic evaluation. Pulmonary parenchyma often presents consolidations, nodules, cavitations, and other interstitial processes. Airway involvement may include segments of stenosis, intraluminal thickening, edema, ulceration, and hemorrhage. While flexible bronchoscopy is an essential diagnostic tool in the evaluation of respiratory tract involvement in GPA, it can also pose significant risks in patients with severe airway compromise [11]. Early diagnosis and prompt initiation of immunosuppressive therapy are crucial to prevent disease progression and minimize organ damage.

This case highlights the importance of considering GPA in the differential diagnosis of patients with unexplained respiratory symptoms, even in the absence of classical clinical features. The patient had an undiagnosed GPA with severe pulmonary and airway involvement. The unexpected severe glottic edema and reduced vocal cord mobility led to a challenging airway and ultimately resulted in cardiac arrest. This emphasizes the severity of the potential for rapid deterioration and life-threatening airway compromise in patients with GPA. Teamwork, effective communication, and prompt intervention were crucial to a positive outcome.

## Conclusions

This case report presents a rare and challenging situation of undiagnosed GPA presenting with unexpected severe glottic edema during flexible bronchoscopy. The patient's initial presentation with pneumonia-like symptoms masked the underlying autoimmune disease, highlighting the importance of maintaining a broad differential diagnosis in patients with pulmonary and airway involvement. The life-threatening airway compromise during the procedure underscores the potential for rapid deterioration in GPA patients, even in the absence of overt respiratory distress symptoms.

This case emphasizes the critical need for a careful airway assessment and vigilant monitoring during any airway intervention in patients with suspected or confirmed GPA. A coordinated multidisciplinary approach, including experienced anesthesiologists, timely intervention, and effective communication among healthcare providers, is crucial to prevent and manage life-threatening complications and ensure optimal patient outcomes.

## References

[1] Jennette JC, Falk RJ, Bacon PA (2013). 2012 revised International Chapel Hill Consensus Conference nomenclature of vasculitides. Arthritis Rheum.

[2] Kitching AR, Anders HJ, Basu N (2020). ANCA-associated vasculitis. Nat Rev Dis Primers.

[3] Granel J, Korkmaz B, Nouar D, Weiss SA, Jenne DE, Lemoine R, Hoarau C (2021). Pathogenicity of proteinase 3-anti-neutrophil cytoplasmic antibody in granulomatosis with polyangiitis: implications as biomarker and future therapies. Front Immunol.

[4] Seo P, Stone JH (2004). The antineutrophil cytoplasmic antibody-associated vasculitides. Am J Med.

[5] Gómez-Puerta JA, Hernández-Rodríguez J, López-Soto A, Bosch X (2009). Antineutrophil cytoplasmic antibody-associated vasculitides and respiratory disease. Chest.

[6] Hoffman GS, Kerr GS, Leavitt RY (1992). Wegener granulomatosis: an analysis of 158 patients. Ann Intern Med.

[7] Alaani A, Hogg RP, Drake Lee AB (2004). Wegener's granulomatosis and subglottic stenosis: management of the airway. J Laryngol Otol.

[8] Coutinho ML, Portela e Silva R, Oliveira J, Almeida V (2024). Undiagnosed Wegener’s granulomatosis with severe pulmonary and airway involvement. Eur J Anaesthesiol.

[9] Blackabey V, Gan RW, Buglass H, Kaul V, Ward VM (2018). Granulomatosis with polyangiitis causing subglottic stenosis: two cases and their management. AME Case Rep.

[10] Hunter RW, Welsh N, Farrah TE, Gallacher PJ, Dhaun N (2020). ANCA associated vasculitis. BMJ.

[11] Polychronopoulos VS, Prakash UB, Golbin JM, Edell ES, Specks U (2007). Airway involvement in Wegener's granulomatosis. Rheum Dis Clin North Am.

